# Starting an Advanced Practice Provider Professional Writing Group

**Published:** 2017-07-01

**Authors:** Lydia T. Madsen, Kristen Starnes-Ott

**Affiliations:** Department of Acute & Continuing Care, UTHealth School of Nursing, Houston, Texas

A work-related professional writing group presents the opportunity for would-be authors to focus on expertise while fostering collegiality and productivity among colleagues. Across many disciplines, including nursing, the routine practice of individual writing and then meeting as a group to review and/or discuss concepts, progress, and obstacles has been beneficial (Boice, 1990, 1992). The development that can occur with writing groups can include facilitation of progress and encouragement in the professional and/or academic writing process, which can be difficult for a new writer (Boice, 1989; Gray, 2010). 

## ESTABLISHING A WRITING GROUP

Several factors should be considered prior to organizing a writing group. Reeves’s (2002) recommendations for the organizer are summarized in Table 1. 

**Table 1:**
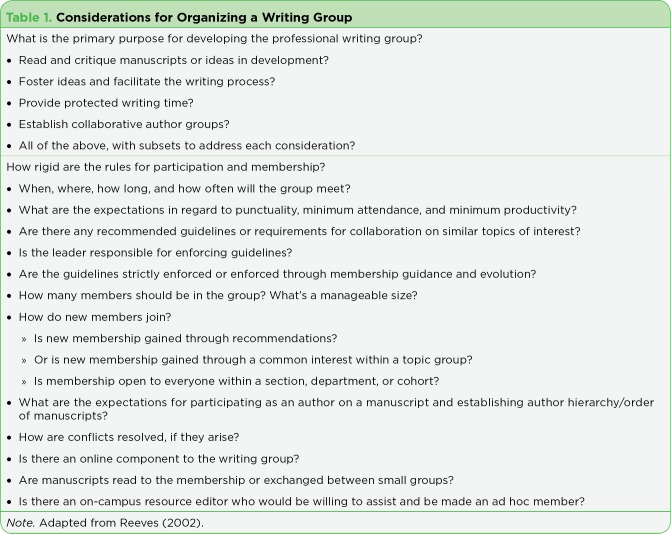
Considerations for Organizing a Writing Group

When the structure of the professional writing group has been outlined, the next step is to identify possible participants. When extending an invitation to join, the basic guidelines for developing a work-related professional writing group (Reeves, 2002; Rosenthal, 2003) are listed in Table 2.

**Table 2:**
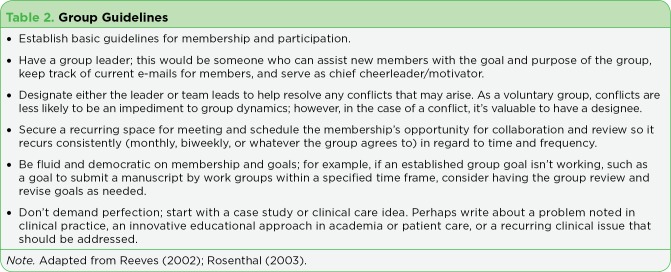
Group Guidelines

## EXEMPLARS OF ADVANCED PRACTICE PROVIDER WRITING GROUPS

Writing groups can take on a variety of members and focus within one particular specialty while serving as editors/coaches for one another (Rickard et al., 2009). For example, a writing group within a large health science center oncology specialty training hospital was established as part of an advanced practice registered nurse (APRN) postgraduate oncology fellowship program. This group formed with the intention to foster and guide ideas in development, facilitate the organization and writing process, and provide critique and editorial assistance with manuscripts as they evolved.

The organization of the writing group was a collaborative effort: The director and associate director of the fellowship program, already established authors, routinely met with the fellows as a small group. The small-group setting served to guide the identification of a topic of interest from the clinical practice of each fellow and subsequently provide extensive input and editing during the development of their individual manuscripts. The end goal of the writing group was each APRN fellow’s completion of the fellowship program with a peer-reviewed publication. Goal success was reflected in the subsequent acceptance and publication of eight of the nine manuscripts submitted after the writing initiative began.

Alternately, another example of a specialty writing group is found in a clinical and educational division at a large health science center school of nursing. The division alternates a writing group leader for a period of time, in this case, an academic year. The group leader is charged with identifying two or three topics for the group to write about for that period. Topics could include recent changes in the specialty program’s curriculum, innovations in clinical education or applied clinical practice, or pilot work in the division’s simulation lab. Along with the topical areas, the leader identifies two journals to target for the completed manuscripts. 

The leader sends out three or four identified topics to the group membership, with members voting on the top two areas to focus the group’s writing efforts for that year. After the topical decisions are made, the leader puts a brief outline together and assigns the various sections of the outline to different writers in the group. Achievable but firm deadlines are determined based on the semester calendar, with drafts of each assigned section sent to the writing group leader. The leader is responsible for assembling the sections and conducting the first draft of editing. The first draft is then sent back to the group membership for both editing and "contextual fit," which is important when multiple writers are involved. Once the drafts are satisfactory to the group, a resource editor assists with final draft edits. The leader or primary author (depending on the topic) is responsible for submitting the manuscript to the targeted journal.

Authorship and overall content agreement should be discussed at the onset of a writing group manuscript. It is helpful if the roles are rotated, so everyone has "ownership" of the process and benefits from the group over time. In the previous example, many of the ideas were driven by continuation of a group member’s doctoral work, clinical area of interest, or the program’s changing curriculum. Primary authorship was based upon this as well and was not always based on who the group leader was. 

## CONCLUSION

Rosenthal (2003) notes that writing groups flourish and are sustained based on the continued value to the members. As noted in the literature and the authors’ experiential exposure to writing groups, cohesion grows from positive experiences within the group. Celebrating an individual’s or a subset group’s acceptance of a manuscript for publication, organizing a group or panel discussion that outlines a variety of specific journal requirements, or presenting a group project abstract at a professional meeting are all opportunities to engage, highlight success, and keep the group relevant to members’ needs.

The value in writing as a professional is often overlooked and understated. An individual’s publication of scholarly writing has historically been attributed to powerful mentors or personal initiative; however, a writing group provides a platform for a systematic approach to scholarship (Jalongo, Boyer, & Ebbeck, 2014). Without publishing clinical observations, educational innovations, evidence-based reviews, and research data, the opportunity to shape policy, identify opportunities for funding research, and initiate change in educational strategies may all go unrecognized (Boice, 1992, p 161). A professional, work-related writing group may provide the initiative for work colleagues to find their voice and share clinical or academic experience with peers.
